# Metabolic and Anthropometric Alterations in Juvenile Idiopathic Arthritis: A Focus on Cardiometabolic Risk and Non-Invasive Evaluation Methods

**DOI:** 10.3390/metabo16020090

**Published:** 2026-01-24

**Authors:** Agnieszka Januś, Justyna Roszkiewicz, Elżbieta Smolewska

**Affiliations:** Department of Paediatric Cardiology and Rheumatology, Medical University of Lodz, 91-738 Lodz, Poland; daniluk.agnieszka1@gmail.com (A.J.); justyna.roszkiewicz@umed.lodz.pl (J.R.)

**Keywords:** juvenile idiopathic arthritis, cardiovascular risk, atherosclerosis

## Abstract

Juvenile idiopathic arthritis (JIA) is the most prevalent chronic rheumatologic condition in childhood, with an incidence that continues to rise worldwide. Despite substantial progress in therapeutic strategies over the past two decades, JIA remains a major health concern. Beyond joint inflammation and musculoskeletal impairment, accumulating evidence indicates that JIA is associated with metabolic disturbances and altered body composition, which may predispose affected children to an elevated cardiovascular risk in the long term. The objective of this review is to synthesize current knowledge on these metabolic and anthropometric alterations and to evaluate the role of non-invasive diagnostic methods in detecting early cardiovascular changes. A narrative review of the literature was conducted using PubMed and Scopus databases, focusing on studies assessing lipid metabolism, insulin resistance, adiposity, and cardiovascular markers in pediatric patients with JIA. Special attention was given to non-invasive diagnostic approaches, including bioelectrical impedance analysis (BIA), dual-energy X-ray absorptiometry (DXA), skinfold thickness, transient elastography, carotid intima–media thickness (cIMT), as well as selected biochemical markers. Evidence suggests that children with JIA frequently present with dyslipidemia, increased insulin resistance, and abnormal body fat distribution compared with their healthy peers. Non-invasive assessment methods, particularly DXA and cIMT, have demonstrated sensitivity in detecting subclinical metabolic and vascular changes. These alterations resemble early features of metabolic syndrome and are thought to contribute to premature cardiovascular morbidity in this population. Incorporating non-invasive cardiovascular risk assessment into routine rheumatology practice may improve early detection of metabolic and vascular complications in JIA, support timely preventive interventions, and ultimately enhance long-term outcomes for affected children. Most available evidence is derived from cross-sectional studies, highlighting the need for longitudinal investigations to better define long-term cardiometabolic risk in JIA.

## 1. Introduction

**Figure 1:**
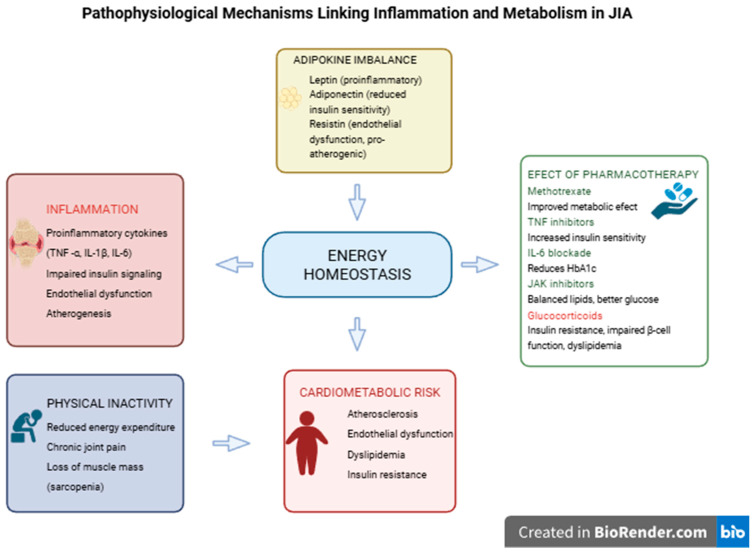
Pathophysiological mechanism linking inflammation in JIA.

Juvenile idiopathic arthritis (JIA) represents a heterogeneous group of inflammatory joint disorders of unknown etiology that occur in children under 16 years of age, with an estimated prevalence of approximately 1 per 1000 children [1]. According to the International League of Associations for Rheumatology (ILAR), JIA comprises seven subtypes that differ in clinical presentation, laboratory findings, disease course, and prognosis: (a) oligoarthritis; (b) rheumatoid factor (RF)-positive polyarthritis; (c) RF-negative polyarthritis; (d) systemic arthritis; (e) psoriatic arthritis; (f) enthesitis-related arthritis; and (g) undifferentiated arthritis [2]. Although the primary manifestation of JIA involves articular symptoms resulting from inflammation and damage to the synovial membrane, cartilage, and periarticular tissues, increasing evidence indicates that systemic inflammation in JIA also contributes to metabolic disturbances. Persistent chronic inflammation, immune dysregulation, pharmacological therapy, and reduced physical activity are key contributors to these metabolic abnormalities [Figure 1]. JIA represents a heterogeneous group of disorders that differ substantially in inflammatory burden, treatment exposure, disease duration, and long-term outcomes. Consequently, metabolic and cardiometabolic risk may not be uniform across JIA subtypes. Systemic and polyarticular forms, characterized by higher inflammatory activity and more intensive pharmacotherapy, may be associated with a greater risk of metabolic alterations compared with oligoarticular disease. This heterogeneity should be considered when interpreting available data and when drawing generalized conclusions.

The aim of this review is to comprehensively examine metabolic and anthropometric alterations observed in children and adolescents with JIA, with particular emphasis on their potential contribution to long-term cardiometabolic risk. Specifically, this review synthesizes current evidence on how chronic inflammation, therapeutic interventions, and lifestyle-related factors interact to influence body composition, lipid metabolism, glucose homeostasis, and vascular function. Furthermore, we aim to evaluate the applicability, accuracy, and clinical relevance of non-invasive and minimally invasive methods used to assess metabolic health and cardiometabolic risk in this population, including anthropometric indices, body composition assessment tools, vascular imaging techniques, and biomarker-based approaches. By integrating available data, this review seeks to identify knowledge gaps, highlight emerging diagnostic strategies, and underscore the importance of early detection and monitoring of metabolic alterations as part of comprehensive JIA management.

This narrative review summarizes available evidence on metabolic and cardiometabolic alterations in children and adolescents with juvenile idiopathic arthritis (JIA). The literature search was conducted primarily in PubMed and Scopus databases and covered studies published approximately between 2000 and 2024. Both pediatric JIA studies and selected adult rheumatoid arthritis (RA) studies were included. Adult RA data were intentionally considered to support mechanistic interpretation in areas where pediatric evidence remains limited, particularly regarding long-term cardiometabolic risk and inflammatory–metabolic pathways. This methodological approach aims to contextualize pediatric findings within the broader framework of chronic inflammatory rheumatic diseases.

## 2. Pathophysiological Mechanisms Linking Inflammation and Metabolism in JIA

### 2.1. Proinflammatory Cytokines and Metabolic Dysregulation

One of the principal mechanisms underlying metabolic dysregulation and cardiometabolic alterations in JIA is the chronic activation of proinflammatory cytokines, including tumor necrosis factor-α (TNF-α), interleukin-1β (IL-1β), and interleukin-6 (IL-6). In pediatric JIA populations, elevated levels of these cytokines have been associated with increased disease activity and systemic inflammation, while mechanistic insights linking cytokine excess to insulin resistance, dyslipidemia, and endothelial dysfunction are largely derived from adult RA cohorts [3,4,5,6,7,8,9].

Studies in adults with rheumatoid arthritis (RA) demonstrate a significant role of proinflammatory cytokines in lipid abnormalities. TNF-α, IL-1β, and IL-6 reduce lipoprotein lipase (LPL) activity, impairing triglyceride clearance and promoting hypertriglyceridemia [10]. Furthermore, chronic inflammation alters hepatic lipid metabolism, leading to decreased high-density lipoprotein cholesterol (HDL-C) levels and modifications of low-density lipoprotein (LDL) particles toward smaller, denser, and more atherogenic forms [11,12].

Chronic inflammation also plays a central role in the initiation and progression of atherosclerotic lesions. In autoimmune diseases, persistent immune activation disrupts endothelial homeostasis and promotes oxidative stress. Proinflammatory cytokines—particularly TNF-α, IL-1β, and IL-6—stimulate the expression of adhesion molecules (VCAM-1, ICAM-1, and E-selectin) on endothelial cells, promoting monocyte adhesion and migration into the vascular intima [13]. Within the infima layer, these monocytes differentiate into macrophages and, upon exposure to oxidized LDL, transform into foam cells—an early hallmark of atherosclerotic plaque formation. Prolonged inflammation fosters smooth muscle cell proliferation and extracellular matrix deposition, ultimately leading to the formation of unstable atherosclerotic plaques that are prone to rupture and thrombosis. Studies in RA patients have confirmed an increased prevalence of pro-atherogenic risk factors and subclinical vascular changes [13,14,15,16].

### 2.2. Adipokines as Mediators of Inflammation and Metabolic Imbalance

Adipose tissue acts as a dynamic endocrine organ that secretes adipokines—bioactive peptides with key roles in metabolism, immune regulation, and vascular homeostasis. In JIA, dysregulation of both pro- and anti-inflammatory adipokines contributes to systemic inflammation, altered body composition, and increased cardiometabolic risk. The best-studied adipokines in this context are leptin, adiponectin, and resistin.

Leptin, predominantly produced by white adipose tissue, exerts pro-inflammatory effects by stimulating T-cell proliferation, enhancing Th1 cytokine production, and suppressing regulatory T-cell activity. Elevated leptin concentrations have been documented in JIA and correlate positively with disease activity, inflammatory markers (CRP, ESR), and body mass index (BMI) [17,18].

In contrast, adiponectin possesses anti-inflammatory and insulin-sensitizing properties. Lower serum adiponectin levels have been associated with insulin resistance, atherogenic lipid profiles, and increased cardiometabolic risk in JIA [19,20]. Resistin further amplifies inflammation by inducing TNF-α and IL-6 production, promoting endothelial dysfunction, and correlating with early atherosclerotic changes such as increased carotid intima–media thickness (cIMT) [19,21,22,23]. Together, these adipokines reflect the intersection of low-grade inflammation and metabolic imbalance more sensitively than traditional inflammatory markers alone.

Clinical studies have yielded partly divergent results, likely due to differences in disease activity, treatment status, and body composition. Eren et al. [17] reported reduced serum leptin and adiponectin concentrations in JIA patients compared with BMI-matched controls. Similarly, Giardullo et al. [24] demonstrated in a systematic review that leptin levels are generally elevated and correlate with disease activity in rheumatic diseases, while adiponectin findings remain inconsistent across subtypes and treatment stages. Conversely, Markula-Patjas et al. [20] found no significant differences in leptin or adiponectin after adjusting for fat mass in glucocorticoid-treated children, suggesting treatment-related modulation.

Evidence also points to dynamic adipokine changes during therapy. Winsz-Szczotka et al. [25] observed that newly diagnosed, untreated JIA patients had elevated adiponectin and resistin but decreased leptin levels relative to healthy peers. Following treatment and clinical remission, some but not all adipokine abnormalities normalized, and these molecules—particularly adiponectin and leptin—correlated with markers of cartilage metabolism, BMI, CRP, and ESR, underscoring their link to both inflammation and tissue remodeling.

Longitudinal data from Siwiec et al. [18] further support the therapeutic restoration of adipokine balance. In 66 children with active JIA treated with the TNF-α inhibitor etanercept, baseline measurements showed elevated adiponectin and decreased leptin compared with 40 controls. After 24 months of therapy, adiponectin levels decreased, leptin normalized, and tenascin C (a marker of extracellular matrix remodeling) also declined, suggesting partial recovery of adipose–immune homeostasis with effective anti-inflammatory therapy.

Collectively, current evidence indicates that adipokines serve not only as markers of systemic inflammation but also as active mediators linking immune dysregulation, metabolic alterations, and vascular risk in JIA. Their measurement may provide additional insight into cardiometabolic complications beyond conventional laboratory indices.

Adiponectin alterations in JIA appear heterogeneous; following treatment and clinical remission, adiponectin levels may increase or normalize, showing inverse correlations with inflammatory markers and BMI, while leptin generally decreases. These correlations indicate a dynamic interaction between adipokines, inflammation, and tissue remodeling.

### 2.3. Effects of Pharmacotherapy on Metabolic Pathways in JIA

Pharmacological treatment plays a crucial role in modifying both inflammation and metabolic balance in JIA. However, anti-inflammatory therapies may exert heterogeneous effects on carbohydrate and lipid metabolism depending on the drug class.

Glucocorticoids profoundly affect glucose and lipid homeostasis by enhancing hepatic gluconeogenesis, promoting protein catabolism, and inducing peripheral insulin resistance [26,27]. Short-term use, such as bridging therapy, may transiently exacerbate insulin resistance, whereas long-term corticosteroid therapy is associated with weight gain, hypertension, and dyslipidemia [28]. Data from adult cohorts with RA indicate that prolonged corticosteroid exposure increases insulin resistance, impairs β-cell function, and raises the risk of diabetes mellitus (DM) [29,30,31]. Risk of DM in adult patients with RA increases with dosage—each 5 mg increase in current oral glucocorticoids was associated with a 25–30% increased risk of DM [31].

In contrast, methotrexate (MTX)—a cornerstone of JIA management—has more favorable metabolic effects. By activating adenosine monophosphate-activated protein kinase (AMPK), MTX enhances glucose uptake, fatty acid oxidation, and mitochondrial biogenesis, thereby improving aspects of metabolic dysregulation such as hyperglycemia and insulin resistance [32]. Moreover, effective disease suppression with MTX has not been associated with deterioration of glucose metabolism and may indirectly improve metabolic control [33].

Biologic and targeted synthetic disease-modifying antirheumatic drugs (bDMARDs and tsDMARDs) exert variable but generally beneficial effects on metabolic parameters. Tumor necrosis factor (TNF)-α inhibitors have been associated with improved insulin sensitivity through downregulation of TNF-driven insulin resistance pathways [33,34,35]. In psoriasis, these agents transiently increase HDL levels [36], while in JIA, 12 months of etanercept therapy resulted in reduced triglycerides and decreased HDL-C, LDL-C, total cholesterol (TC), and atherogenic index among responders [37].

IL-6 receptor blockade with tocilizumab has been shown to enhance insulin sensitivity and reduce markers of insulin resistance in both RA and non-diabetic patients [35,38], although elevations in triglyceride levels have also been reported [39]. In comparative analyses, tocilizumab reduced glycated hemoglobin (HbA1c) more effectively than TNF inhibitors [35].

Finally, Janus kinase (JAK) inhibitors, by modulating the JAK–STAT signaling pathway, influence lipid metabolism and glucose regulation. While pediatric data remain limited, adult RA studies demonstrate increases in both LDL and HDL cholesterol without changes in the LDL/HDL ratio [40,41,42]. Moreover, baricitinib therapy has been associated with improved insulin sensitivity and glucose uptake in RA patients with concomitant type 2 diabetes [43].

In summary, pharmacotherapy in JIA has complex metabolic implications. Glucocorticoids tend to exacerbate insulin resistance and dyslipidemia, whereas methotrexate, TNF inhibitors, and IL-6 or JAK blockade may partially reverse inflammation-driven metabolic disturbances. Biologic therapies and Janus kinase inhibitors may induce changes in lipid profiles; however, these changes likely reflect effective inflammation control rather than true metabolic deterioration. Importantly, long-term cardiovascular outcome data in pediatric populations remain unavailable.

### 2.4. Physical Inactivity and Metabolic Risk in JIA

Reduced physical activity in JIA contributes mechanistically to metabolic risk by promoting insulin resistance, unfavorable body composition, and persistence of low-grade inflammation. Thus, physical inactivity should be considered a modifiable component within the pathophysiological cascade linking inflammation and metabolic dysregulation. Pain, joint stiffness, and deformities limit spontaneous movement and decrease energy expenditure, leading to muscle atrophy (sarcopenia) and increased visceral adiposity [44,45,46,47]. These changes in body composition exacerbate insulin resistance and dyslipidemia.

Moreover, even JIA patients in remission exhibit lower lipid oxidation rates during exercise compared to healthy peers [48].

Nesbitt et al. [49] reported reduced daily moderate-to-vigorous physical activity in children with JIA compared with typically developing peers, identifying physical limitations, disease-related symptoms, pain and fatigue, exercise-induced discomfort, social concerns, and the avoidance of activity as a conditioned behavioral response among the contributing factors.

Persistent inactivity during childhood and adolescence is associated with an increased likelihood of developing an unfavorable cardiometabolic profile in adulthood; however, even modest increases in physical activity can mitigate this risk. Exercise interventions—including both resistance and aerobic training—as well as treatments that reduce systemic inflammation (TNF blockade) have been shown to improve muscle metabolism and partially restore fat oxidation during exercise [50,51]. Thus, structured physical activity represents a key component of cardiometabolic risk-reduction strategies in patients with JIA.

## 3. Laboratory and Clinical Evidence of Metabolic Alterations

The pathophysiological pathways outlined above translate into clinically detectable metabolic and anthropometric abnormalities in children and adolescents with JIA. The following section summarizes available clinical evidence demonstrating how chronic inflammation, altered adipokine profiles, and therapeutic exposures manifest as measurable metabolic alterations.

### 3.1. Lipid Profile and Apolipoproteins

Recent evidence indicates that JIA is associated with multiple lipid abnormalities. A systematic meta-analysis including 16 studies and 1502 participants (639 JIA patients and 863 healthy controls) demonstrated that JIA patients exhibit reduced levels of HDL cholesterol and VLDL cholesterol, alongside elevated LDL cholesterol. In the same analysis, apolipoprotein A-I (apoA-I)—the major structural component of HDL—was markedly decreased in JIA [52]. These results suggest the presence of a proatherogenic lipid pattern that may increase the risk of future cardiovascular events.

A cross-sectional study involving 62 JIA patients revealed dyslipidemia in 83.3% of participants, with low HDL-C being the most frequent abnormality. Lipid disturbances varied among subtypes, with patients with systemic JIA showing higher frequencies of abnormal LDL-C, apolipoprotein B (ApoB), and non-HDL-C levels compared with those with polyarticular JIA. Importantly, ApoA-I concentrations negatively correlated with ESR, indicating that systemic inflammation directly contributes to lipid abnormalities. Furthermore, the study reported that patients receiving biologic therapy had higher ApoA-I concentrations, suggesting a beneficial effect of targeted treatment [53].

Similarly, Marangoni et al. [54] reported dyslipoproteinemia in 71% of polyarticular JIA patients, with 57% showing decreased HDL-C levels, confirming that this alteration is one of the most consistent metabolic findings in JIA. A retrospective analysis of 186 children and adolescents with autoimmune rheumatic diseases (including JIA) identified dyslipidemia in 68.8% of subjects, most commonly characterized by reduced HDL-C (39.8%) [55]. Disease activity and treatment efficacy appear to significantly influence lipid profiles, as studies have demonstrated improvements in serum lipids and atherogenic indices following effective anti-rheumatic therapy [56].

Long-term follow-up studies in adults with a history of JIA have shown persistently elevated total and LDL cholesterol levels, along with reduced HDL-C [57]. Taken together, JIA is associated with a distinct dyslipidemic profile characterized by reduced HDL-C and ApoA-I and elevated LDL-C levels. These changes are largely driven by inflammation and can be detected even in children with normal body weight, suggesting that systemic immune activation—rather than obesity—underlies the observed lipid abnormalities. The presence of these changes may therefore serve as early biochemical indicators of a proatherogenic state.

### 3.2. Glucose Metabolism and Insulin Resistance

Children and adolescents with JIA frequently exhibit disturbances in glucose homeostasis and early insulin resistance, which may precede overt hyperglycemia and contribute to long-term cardiometabolic risk. In a study involving non-obese, prepubertal children with JIA and matched healthy controls, oral glucose tolerance test (OGTT) results did not differ significantly; however, fasting insulin levels and indices of insulin resistance were markedly elevated in the JIA group, suggesting early metabolic impairment [58]. Another study examining systemic JIA (sJIA) patients across different BMI categories found that obese sJIA children exhibited reduced insulin sensitivity and increased insulin secretion compared with BMI-matched healthy peers [27].

Studies conducted in adults with RA have yielded similar findings. The prevalence of insulin resistance is higher in patients with RA than in the general population [59,60,61]. Moreover, a positive association has been demonstrated between disease activity and the homeostatic model assessment of insulin resistance (HOMA-IR) index. Shahin et al. [62] reported that insulin resistance was more pronounced in patients with high disease activity compared with those with moderate disease activity.

Clinically, laboratory abnormalities such as elevated fasting insulin, increased HOMA-IR, reduced insulin sensitivity indices, or impaired OGTT results translate into a higher likelihood of visceral adiposity, endothelial dysfunction, proatherogenic lipid changes, and ultimately, accelerated atherogenesis if these alterations persist.

## 4. Anthropometric and Body Composition Changes

### 4.1. Anthropometric Indicators

BMI, derived from height and weight measurements, remains the simplest and most accessible tool for anthropometric assessment. Several studies have demonstrated that obesity, as determined by BMI, is a significant predictor of poorer disease control at six months in JIA patients [63]. A Nordic cohort study of young adults with JIA revealed associations between BMI, health-related quality of life, disease activity, and disability. Similarly, a Portuguese–Brazilian analysis identified underweight status as independently associated with higher disease activity [64]. Gicchino et al. [65] further emphasized that both underweight and obesity may negatively affect disease progression. Despite its utility, BMI fails to distinguish between fat and lean mass, potentially overlooking sarcopenia or excess adiposity in individuals with normal weight.

### 4.2. Body Composition Analysis

Several methods are available for the assessment of body composition and adiposity, including skinfold (SF) measurements, bioelectrical impedance analysis (BIA), and dual-energy X-ray absorptiometry (DXA). Although SF and BIA are inexpensive, their accuracy may be limited in children with chronic diseases [66] or excessive adiposity [67]. Comparative analyses in JIA patients indicate that BIA underestimates fat mass and overestimates lean mass relative to DXA, both in patients and controls [68].

JIA patients tend to exhibit a higher body fat percentage than healthy peers, and even those with low disease activity may display increased central and peripheral adiposity [69]. However, a Spanish study found no significant differences in BMI or body composition between well-controlled JIA patients and healthy controls [70], consistent with findings from a 2020 systematic review [71].

Body composition assessment is also valuable for evaluating bone and muscle mass, enabling early detection of osteoporosis and sarcopenia. Studies by Wiech et al. [72] and Jednacz et al. [73] demonstrated reduced muscle mass in girls with JIA, particularly those with the polyarticular subtype.

## 5. Non-Invasive Assessment of Cardiometabolic Risk

### 5.1. Carotid Intima–Media Thickness and Vascular Changes

Non-invasive assessment tools differ in their applicability to routine clinical practice versus research settings. While anthropometric indices and standard biochemical parameters are readily feasible in daily care, advanced vascular imaging techniques such as carotid intima–media thickness (cIMT) should be interpreted cautiously in JIA, as their prognostic value for future cardiovascular events in pediatric populations remains uncertain. Carotid intima–media thickness is one of the most widely used non-invasive markers of subclinical atherosclerosis and has been employed to detect early vascular changes in children and adolescents with chronic inflammatory diseases, including JIA.

Patients with JIA exhibit increased atherogenicity resulting from chronic immune activation and inflammation-induced metabolic disturbances. Chronic inflammation leads to endothelial dysfunction through increased expression of adhesion molecules, elevated production of proinflammatory cytokines, and oxidative stress [23,74]. These mechanisms disrupt the balance between vasodilatory and vasoconstrictive factors, promoting the early development of atherosclerotic lesions. Furthermore, active inflammation affects lipid metabolism, resulting in so-called inflammatory dyslipidemia: decreased HDL cholesterol levels and impaired antioxidant capacity, alongside an increase in small, dense LDL particles with higher atherogenic potential [75]. These interacting processes promote intimal lipid deposition, inflammatory cell recruitment, smooth muscle cell proliferation, and extracellular matrix remodeling—structural changes detectable as increased cIMT on high-resolution ultrasound.

Hussain et al. [76] reported significantly higher cIMT and reduced brachial artery flow-mediated dilation (FMD) in patients with active versus inactive disease, with correlations between cIMT and blood pressure, total and LDL cholesterol, and inverse associations with HDL cholesterol. Similarly, Del Giudice et al. [77] demonstrated increased carotid and aortic intima–media thickness (aIMT) in pediatric rheumatic disease cohorts. Ilisson et al. [21] found increased cIMT and higher myeloperoxidase levels in children with newly diagnosed JIA, suggesting that both structural and oxidative mechanisms operate early in the disease course. Breda et al. [78] reported associations between proinflammatory cytokines, oxidative stress markers, and higher cIMT in children with JIA, and observed that some vascular indices improved with treatment over time. Together, these studies imply that active, untreated, or long-standing inflammation amplifies vascular remodeling detectable by cIMT.

On the other hand, several studies have failed to demonstrate consistent increases in cIMT across all JIA cohorts. Mani et al. [79] reported no significant difference in cIMT between JIA patients and healthy controls despite marked HDL dysfunction, indicating that lipoprotein functional changes may precede measurable arterial wall thickening in some populations.

### 5.2. Hepatic Steatosis and Transient Elastography Findings

Hepatic steatosis, or non-alcoholic fatty liver disease (NAFLD), is increasingly recognized in patients with chronic inflammatory conditions, including RA. A systematic analysis of individuals with RA estimated that the prevalence of NAFLD may reach approximately 35% [80]. A meta-analysis updated in 2025 reported a prevalence of about 22.8% for NAFLD/NASH among RA patients, with substantial variability depending on the diagnostic modality—for instance, transient elastography identified steatosis in up to 33.3% of cases [81]. Ultrasonographic assessments have similarly shown moderate to severe hepatic steatosis in nearly 38.5% of RA patients (compared with ~19.8% of healthy controls), with higher BMI, male sex, and elevated triglyceride levels emerging as independent predictors of liver involvement [82].

Recent studies have also underscored the relevance of hepatic alterations in juvenile idiopathic arthritis. In a cohort of JIA patients treated with MTX, 21.7% demonstrated hepatic steatosis on transient elastography, while no cases of fibrosis were detected. Overweight status, obesity, and greater cumulative methotrexate exposure were significantly associated with steatosis in this population [83].

Collectively, these findings indicate that hepatic steatosis is relatively common in both JIA and RA. Transient elastography offers a valuable, noninvasive method for early detection, and in children with JIA it may help identify those at increased risk—particularly individuals with excess body weight or substantial cumulative exposure to MTX.

## 6. Conclusions and Future Perspectives

Children and adolescents with JIA are at heightened risk for metabolic and cardiovascular disturbances, including dyslipidemia, insulin resistance, adipokine imbalance, and altered body composition. These abnormalities may persist into adulthood, contributing to premature cardiovascular morbidity. Chronic inflammation, high disease activity, and anti-inflammatory pharmacotherapy are key contributors to these metabolic derangements.

Metabolic alterations in JIA arise from a complex and multifactorial interplay between chronic inflammation, disease heterogeneity, pharmacological treatment, and lifestyle factors. Cardiometabolic comorbidity clustering may identify subsets of patients with higher inflammatory burden and potentially more aggressive disease phenotypes, as demonstrated in adult RA populations. Ruscitti et al. [84] indicate that in patients with RA, cardiometabolic multimorbidity identifies a group of patients with a severe form of the disease and a higher inflammatory burden, while Conforti et al. [85] indicate a higher risk of venous thromboembolism in patients with RA, probably related to the interaction between systemic inflammation and coexisting cardiovascular diseases. Although such data cannot be directly extrapolated to JIA, they provide a useful conceptual framework.

Chronic inflammation appears to represent a unifying mechanism linking immune dysregulation, endocrine disturbances, and early atherosclerotic changes. While cardiometabolic abnormalities are often subtle or subclinical during childhood, persistent inflammation may accelerate atherosclerosis and increase cardiovascular risk later in life [86]. An important issue concerning JIA is the coexistence of hormonal and metabolic disorders, such as Cushing’s syndrome (CS), Hashimoto’s disease (HD), type 1 diabetes (T1D), growth disorders and hypothalamic–pituitary–adrenal (HPA) axis disorders, which may also increase cardiovascular risk [87].

Important knowledge gaps remain, particularly regarding longitudinal pediatric data, subtype-specific risk stratification, and long-term cardiovascular outcomes.

Early recognition and monitoring of cardiometabolic risk factors should therefore become an integral part of JIA management. Regular assessment of lipid profiles, glucose metabolism, and body composition, combined with lifestyle interventions and optimization of anti-inflammatory therapy, may help mitigate long-term complications. Future longitudinal studies are needed to clarify causal mechanisms and establish evidence-based guidelines for screening and prevention of cardiometabolic comorbidities in JIA.

## Data Availability

All data relevant to the study are included in the article.
